# Current insights of applying MRI in Graves’ ophthalmopathy

**DOI:** 10.3389/fendo.2022.991588

**Published:** 2022-09-29

**Authors:** Cheng Song, Yaosheng Luo, Genfeng Yu, Haixiong Chen, Jie Shen

**Affiliations:** ^1^ Department of Endocrinology and Metabolism, Shunde Hospital of Southern Medical University (The First People’s Hospital of Shunde), Foshan, China; ^2^ The Second School of Clinical Medicine, Southern Medical University, Guangzhou, China; ^3^ Department of Radiology, Shunde Hospital of Southern Medical University (The First People’s Hospital of Shunde), Foshan, China

**Keywords:** Graves ophthalmopathy, MRI, orbital fat, extraocular muscles, assessment

## Abstract

Graves’ ophthalmopathy (GO) is an autoimmune disease related to Grave’s disease (GD). The therapeutic strategies for GO patients are based on precise assessment of the activity and severity of the disease. However, the current assessment systems require development to accommodate updates in treatment protocols. As an important adjunct examination, magnetic resonance imaging (MRI) can help physicians evaluate GO more accurately. With the continuous updating of MRI technology and the deepening understanding of GO, the assessment of this disease by MRI has gone through a stage from qualitative to precise quantification, making it possible for clinicians to monitor the microstructural changes behind the eyeball and better integrate clinical manifestations with pathology. In this review, we use orbital structures as a classification to combine pathological changes with MRI features. We also review some MRI techniques applied to GO clinical practice, such as disease classification and regions of interest selection.

## Introduction

Graves’ ophthalmopathy (GO) is an extrathyroidal manifestation of Graves’ disease (1). Approximately 20%–30% of GD patients suffer from GO, and it is more common in women. The prevalence of GO is reported to be between 90 and 155 per 100,000 people in Europe and 100–300 per 100,000 in Asia (2, 3). Although the incidence rate of GO is relatively low, it has a significant impact on the quality of life of patients, whether in mental health or socio-economic status (4). Various clinical presentations can be observed in GO, including proptosis, eyelid retraction, periorbital tissue edema, and compressive optic neuropathy. Therefore, accurate treatment is important to improve the symptoms of patients. Despite the vast progress made in the understanding of GO pathogenesis, treating the condition can still be problematic. The management of GO depends on an accurate assessment of its severity and activity. Symptom-and-sign-based systems, such as Clinical Activity Scores (CAS), classifications by the European Group on Graves’ Orbitopathy (EUGOGO), NOSPECS, and VISA (vision, inflammation, strabismus, and appearance), have been widely accepted to assess GO severity and activity (5). However, these classifications can be subject to clinical experience and patient status, and more objective assessments are needed.

Magnetic resonance imaging (MRI) is a non-invasive medical imaging method. It has long been applied in GO assessment and differential diagnosis, which is non-radiation and provides high resolution in soft tissue (6). Recently, major progress has been made in MRI for GO. This review summarizes the application of MRI sequences to different tissues involved in GO. We have compared the efficacy of these sequences in view of more objective prediction and diagnosis to assist physicians in selecting better protocols.

## Pathogenesis of GO

It has been postulated that the thyroid-stimulating hormone receptor (TSHR) is the primary potential target for GO initiation. Recent studies have suggested that the insulin-like growth factor 1 receptor (IGF-1R) also plays a critical role in GO development (7). Orbital fibroblasts (OFs), which express TSHR and/or IGF-1R, are activated to secrete pro-inflammatory factors and extraocular matrix (ECM). Meanwhile, other immune cells such as T cells, B cells, and monocytes are mobilized *via* chemotaxis to reach retrobulbar tissue, forming an orbital inflammatory microenvironment (8, 9). In the early stage, T helper (Th) 1 cells play a major role, secreting IL-1β, IL-2, IFN-γ, etc. These pro-inflammatory cytokines promote the proliferation of OFs, accelerate the production of glycosaminoglycans, and induce the differentiation of OFs. Regarding the inactive or late phase, activation of Th2 cells leads to anti-inflammatory cytokines secretion, with representative as IL-4 and TGF-β, which promote tissue repairment (10). Th17 also serves as a critical cell, contributing to inflammation and fibrosis (11). As a result, the orbital fat expands, which results in overt exophthalmos. The extraocular muscles (EOMs) become swollen and suffer from limited motility, leading to diplopia or strabismus. This inflammatory microenvironment and ECM accumulation is associated with periorbital edema. In severe cases, the crowded orbit increases the mechanic pressure, exacerbating pain or even compressing the optic nerve and veins (8, 12, 13).

## Why we choose MRI?

A small proportion of GO cases do not present with thyroid dysfunction, and, there are many alternative conditions that might mimic GO, such as idiopathic orbital inflammation, sarcoidosis, Sjogren syndrome, and vasculitis (14). Moreover, as aforementioned, soft tissues such as EOMs and orbital fat are involved in GO, and their pathology reflects the status of the disease. Thus, a comprehensive supplementary examination is necessary to help physicians identify GO from other diseases and classify GO more accurately.

Pathological biopsy provides the most accurate method for early diagnosis and staging of GO. However, this procedure presents a relatively high risk of side effects and suffers from low adherence, so it is problematic to promote. Another approach that could provide precise information is imaging, including computed tomography (CT), MRI, and ultrasound examinations. These have the advantages of being non-invasive and time-saving and provide the ability to detect subtle lesions in the retrobulbar structures, so imaging in GO diagnosis is now a research focus. The advantages and disadvantages of these methods are summarized in **Table 1**.

**Table 1:** Comparison of three imaging modalities.

	Ultrasound	CT	MRI
**Morphological changes in orbits**	Medium, especially in blood flow	Strong, especially in bone	Strong, especially in soft tissue
**Assessment for activity**	Weak	Weak	Strong by multiple parameters
**Treatment response monitoring**	Weak	Medium	Strong
**Examination time**	Time-saving	Time-saving	Time-consuming
**Cost**	Price-friendly	Medium	Expensive
**Radiation**	No	Yes	No
**Availability and convenience**	Strong	Medium	Weak
**Contraindications**	–	Pregnancy	Claustrophobia, electronic or magnetic metal implanted

CT, computed tomography; MRI, magnetic resonance imaging.

**Figure 1:**
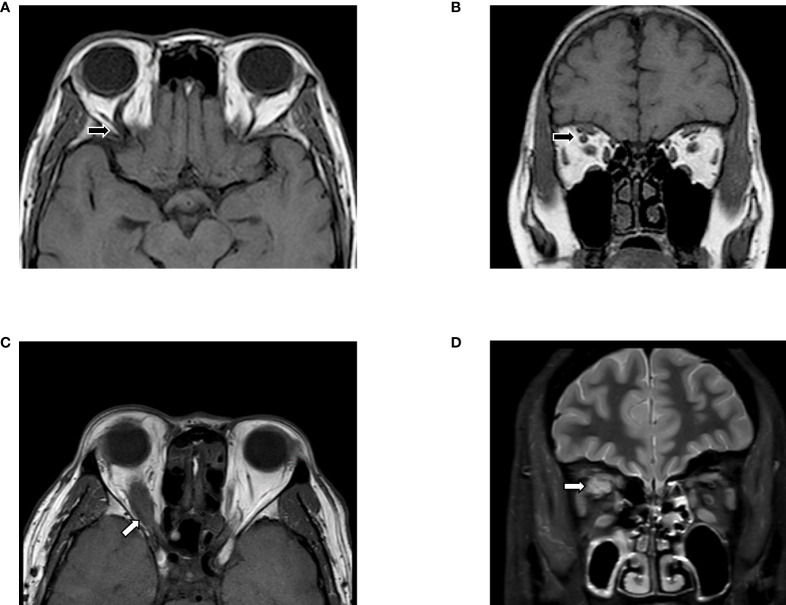
Differential diagnosis based on MRI. **(A, B)** Slightly enlarged EOMs were shown in T1WI, with the unilateral, augmented superior ophthalmic vein (black arrows). Features indicated carotid-cavernous fistulas instead of GO, which need to confirm *via* digital subtraction angiography (DSA). **(C, D)** MRI images suggested Imbalanced exophthalmos and the apparent swelling of superior rectus in the right eye. Meanwhile, in T2FS, increased signal of EOMs, combined with the tendon involved (white arrows) enlargement in superior rectus suggested orbital myositis. The figure is original.

Despite some deficiencies, the advantages of MRI compared to CT or ultrasound are still remarkable, such as the lack of radiation, the high soft tissue resolution, the ability to perform multi-parametric imaging and post-processing, which has resulted in more attention from physicians for this procedure. The differential diagnosis according to symptoms and MRI findings is indicated in **Table 2**, and two cases mimicking GO are depicted in **Figure 1**. The basis for evaluating GO and selecting treatment is complicated pathology, and lots of pathological changes can be captured on MRI, including inflammation, steatosis, and fibrosis.

**Table 2:** Differential diagnosis of GO.

	GO	Orbital lymphoma	IgG4 related ophthalmopathy	Idiopathic orbital inflammation	Carotid-Cavernous Fistulas
Sex distribution	Female	Male	No difference	No difference	Male
Thyroid Dysfunction	Always	Rarely	Rarely	Rarely	Rarely
Increased IgG4	Slightly	Rarely	Obviously	Rarely	Rarely
Clinical manifestations					
Bilateral	Frequently	Rarely	Frequently	Sometimes	Rarely
Pain	Frequently	Sometimes	Rarely	Frequently	Sometimes
Eyelid swelling	Frequently	Rarely	Frequently	Frequently	Rarely
Multiple organs involvement	Always, such as thyroid and pretibial myxedema	Frequently, such as periorbital bone	Always, such as salivary gland and pancreas	Rarely	Rarely
Proptosis	Frequently	Frequently	Frequently	Rarely	Frequently
Conjunctiva involvement	Frequently	Sometimes	Rarely	Frequently	Frequently
MRI features					
Extraocular muscle enlargement	Frequently, without tendon involved	Rarely	Sometimes, tendon can be involved	Sometimes, often in medial muscle, tendon can be involved	Frequently, multiple muscles
Lacrimal gland enlargement	Frequently	Frequently	Always	Sometimes	Rarely
Nerve involved	Sometimes, optic nerve compression	Sometimes, optic nerve compression	Rarely	Rarely	Rarely
Character of lesion on MRI	Active phase: T2WI ↑ Inactive phase: T1 T2 WI –/↓	T1WI – T2WI –/↓ with irregular margin	T1WI – T2WI –/↓ with homogenous and well-defined	Similar to GO	Enlargement and internal signal void of cavernous sinus on T1WI and T2WI

GO, Graves ophthalmopathy; IGG4, immunoglobulin G4; MRI, magnetic resonance imaging; T1WI, T1 weighted-image; T2WI, T2 weighted image. ↑, signal increased; ↓, signal decreased.

## MRI for evaluation of GO

GO is a multi-stage disease in which multiple tissues are involved, causing variable morphological and histological changes in these tissues. As shown in **Table 3**, we briefly describe the anatomy and histology of these tissues in relation to the disease and summarize relevant MRI sequences based on the different target tissues.

**Table 3:** MRI sequences applied in GO assessment.

Tissue or organs	Index	Method	MRI sequence	MRI findings	Reference
Orbital fat	Exophthalmos	the perpendicular distance between the interzygomatic line and the surface of the cornea	T1WI	1–2 mm difference between MRI and Hertel ophthalmometry	Cevik et al. (15) Maria et al. (16)
	Volume	ROI outlined and restructured by Mimics	T1WI with thin layers	Orbital fat volume in GO is higher than healthy control	Shen et al. (17)
	Thickness	The maximum distance between the eyeball and medial wall	T1WI	The thickness increased successively among the healthy control, responsive group and unresponsive group	Hu et al. (18) Xu et al. (19)
EOMs	Diameters	Short Diameter: medial and lateral rectus muscles were measured on axial images, others on coronal images	T1WI	Affected by many factors, a possible predictor of glucocorticoid response	Xu et al. (19)
	Volume	ROI outlined and restructured by Mimics	T1WI with thin section	EOMs volume in GO are higher than healthy control	Shen et al. (17)
EOMs	Inflammation	Draw ROI on the maximum EOMs cross-section	T2 mapping	T2RT got from T2 mapping is higher in therapeutic responsive group than unresponsive group	Zhai et al. (20)
		Draw ROI on the muscle with highest signal intensity	STIR-T2WI	SIR is correlate with CAS	Mayer et al. (21, 22)
			Dixon-T2WI	Dixon-T2WI has fewer artifacts and higher efficacy than traditional FS sequences	Ollitrault et al. (23) Chen et al. (24)
			Echo planar DWI, non-EPI DWI	Both sequences can discriminate GO from controls, but non-EPI DWI might have higher efficacy	Politi et al. (25) Feeney et al. (26)
	Fat infiltration	Intramuscular fat quantification by specific calculation	Dixon-T2WI	FF of EOMs in GO is higher than normal	Das et al. (27)
	Fibrosis	Draw ROI of inferior rectus and medial rectus muscles on the maximum cross-section	Non contrast T1 mapping	Although several EOMs show higher signal on FS sequence, decrease in T1 SI predict unresponsible to therapy	Matsuzawa et al. (28)
		Draw ROI of four rectus muscles at muscle belly precontrast and postcontrast	Pre/post contrast T1mapping	ECV is higher and relate to pathological findings in inactive groups	Ma et al. (29)
Lacrimal gland	Herniation	The perpendicular distance between the interzygomatic line and the most anterior tip	T2WI with FS	The herniation value is higher in active and glucocorticoid responsive patients	Gagliardo et al. (30)
	Inflammation	“Hotspot”: ROI which only a little proportion of the whole cross-section placed on the highest SI region	T2WI with FS	SIR is higher in active GO than inactive	Hu et al. (31)
		Draw ROI on the maximum LG cross-section	T2 mapping	T2 value is higher in GO than GD and it’s an independent predictor for the diagnosis of GO	Wu et al. (32)
Optic nerve	DON	Muscle index and T2 value got from four continuous slices and select the most efficacy slice	Dixon-T2WI, T2 mapping	Muscle index and T2 value are higher in DON	Zou et al. (33)
		The optic nerve sheath diameter, optic nerve diameter and optic nerve subarachnoid space got from two continuous slices and select the most efficacy slice	Modified Dixon-T2WI	The optic nerve subarachnoid space is larger in DON than GO and health control	Wu et al. (34)

MRI, magnetic resonance imaging; T1WI, T1 weighted image; ROI, regions of interest; GO, Graves ophthalmopathy; EOMs, extraocular muscles; T2RT, T2 relaxation time; SIR, signal intensity ratio; CAS, clinical activity score; T2WI, T2 weighted images; FS, fat suppressed; DWI, diffusion weighted image; EPI, echo planar imaging; FF, fat fraction; SI, signal intensity; ECV, extracellular volume; LG, lacrimal gland; GD, Graves’ disease; DON, dysthyroid optic neuropathy.

### Orbital fat

#### Anatomy, histology, and pathologic change in GO

**Figure 2:**
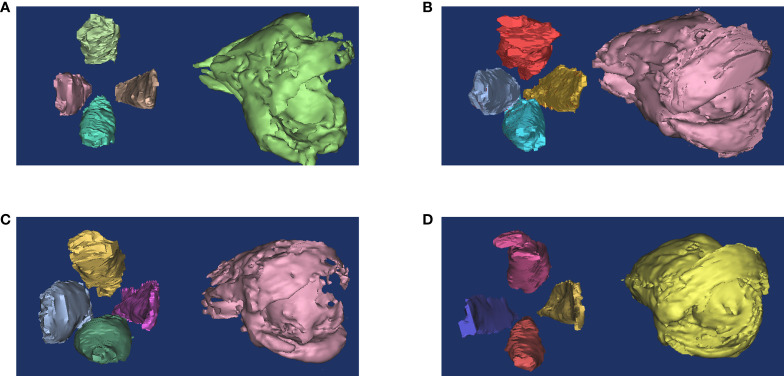
Reconstruction of orbital fat and EOMs. The ratio of orbital fat and EOMs usually change in GO patients. **(A)** Healthy people. **(B)** Both fat and muscle volume increased. **(C)** Muscle volume increased only. **(D)** Fat volume increased only. This figure is original and the classification is based on (39).

About 50% of the orbital volume is formed by orbital fat, which serves to support other structures in the orbit and reduce friction (35) (**Figure 2**). Histologically, orbital adipose tissue can be divided into two types: large adipocytes with thin septa at the orbital apex and small adipocytes with more fibrous septa near the muscles and lacrimal glands (36, 37). To date, there are no studies about whether these two fats have different effects during GO progress. Orbital fibroblasts can differentiate into adipocytes and cause an expansion of fat volume, resulting in a more severe appearance. At the same time, lower orbital fat thickness seems to indicate the better responsive to glucocorticoid (18, 19), despite the relationship between the volume and CAS remains further elucidated (38, 39).

#### Exophthalmos

**Figure 3:**
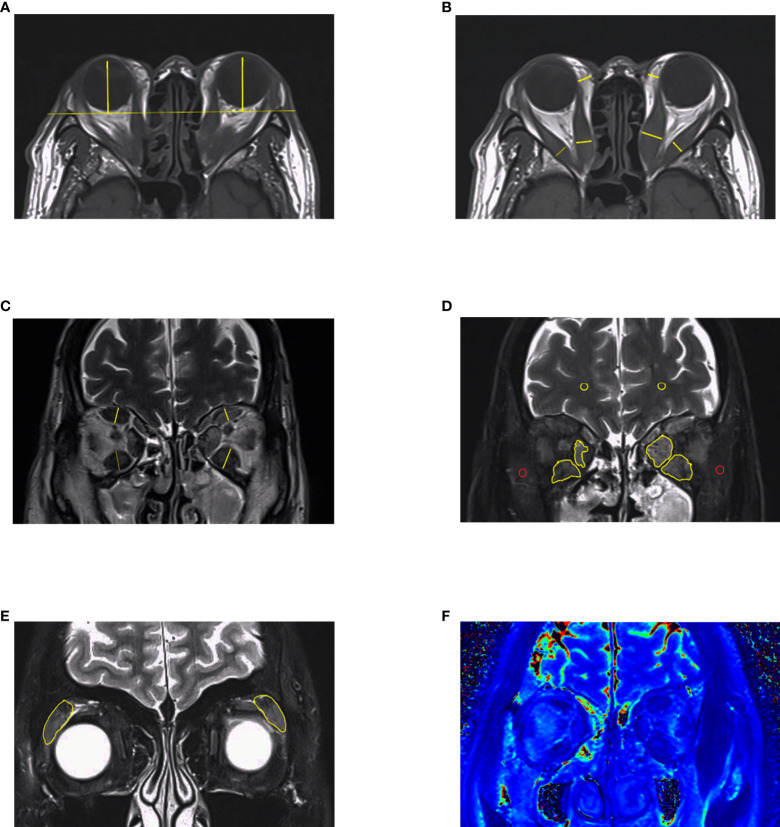
MRI measurements in GO. **(A–C)** Length parameters such as proptosis, thickness of extraocular muscles and thickness of orbital fat. It is noteworthy that thickness of medial rectus and lateral rectus muscles should be measured in axial images. **(D, E)** Signal intensity of extraocular muscles and lacrimal glands got from Dixon-T2WI sequence. The signal intensity of temporalis muscle (red circle) or white matter (yellow circle) on the same slice was used to calculated signal intensity ratio (SIR). **(F)** EOMs displayed on T2 mapping. This figure is original.

Proptosis is a common symptom that occurs in about 60% of GO patients (40). It probably results from the enlargement of adipose tissue, which is the main component of the orbit (39). Exophthalmos, defined as a 3 mm greater than the upper limit of the normal range, contributes to assessing GO “severity” and treatment response (41, 42). However, the relationship between exophthalmos and activity is inexplicit (19, 43, 44). The degree of exophthalmos might be related to various factors, including sex, age, and race (45–49), which is recommended to establish a normal reference in their own area districts. Traditionally, the Hertel exophthalmometer is used to measure exophthalmos. Although it is portable and affordable, accuracy and comparability are limited due to some unavoidable factors, such as the experience of the observers and axial globe position (50, 51). Previous studies have shown that the interclinician reliability of exophthalmos obtained from Hertel ophthalmometry is not as perfect as that measured on imaging (52, 53). For this, the axial slice that most obviously depicts the EOMs and optic nerve is selected, and from which the perpendicular distance between the interzygomatic line and the surface of the cornea is measured (**Figure 3A**) (52). However, depending on the selection of either the anterior or the posterior corneal surface, there can be a difference of 1–2 mm for the exophthalmos determined by MRI and by Hertel ophthalmometry (15, 16). In summary, in the absence of guidance from an experienced ophthalmologist, measuring exophthalmos with MRI is a good diagnostic option.

#### Volume of orbital fat

Orbital fat has an irregular structure and fills the spaces among normal tissues such as nerves, eyeballs, and EOMs, making accurate measurement of the tissue by common MRI difficult (54). Early quantitative measurements were made by subtracting the volume of the six EOMs, the optic nerve, and the eyeball from the entire orbit. However, the fat volume thus derived still included other connective tissues such as the lacrimal gland and blood vessels (55). Another simple method is to measure the thickness of the orbital fat: the distance between the medial wall of the orbit and the medial wall of the eyeball (**Figure 3B**), but the accuracy of these methods is still challenging (19). With the development of three-dimensional technology, software such as MIMICS can measure the volume of orbital fat more accurately *via* reconstruction (**Figure 2**), and ratios of fat volume to orbital bony volume can neutralize gender differences (56). This method has been used for evaluating the therapeutic effect of teprotumumab, but it is widely based on CT rather than MR images (57, 58). However, benefiting from the high resolution of soft tissue, reconstruction with MRI may be more accurate than with CT (17).

### EOMs

#### Anatomy, histology, and pathologic change in GO

Six EOMs enable a wide movement range of the eye, including four recti muscles and two oblique muscles. However, the majority of studies have focused on the morphological and histological changes in the rectus muscles. Compared to limb skeletal muscles, EOMs have a more random arrangement of myogenic fibers and more variation in size. Pathologies such as fiber hypertrophy and myopathy can frequently be observed even in normal EOMs. Additionally, the EOMs contain more mitochondria and have a greater oxidative capacity than other skeletal muscles (59). These factors may have some impact on quantitative MRI. Future studies are needed to establish the baseline of EOMs in healthy subjects (60, 61).

Pathological changes in EOMs are common in GO, with approximately 70% of patients involved (39). These lesions involving the muscle belly are roughly consistent with the course of GO, and quantifying the extent of these lesions may be complementary indicators for assessment, including classification and prediction.

#### Morphological parameters of EOMs

The active phase of GO is usually accompanied by inflammatory edema, leading to changes in several measurable parameters of the EOMs (62). MRI can clearly show EOMs and further measure their diameter, cross-sectional area, and volume. Due to edema in EOMs during the active phase, the short diameter (thickness) measured from coronal MRI is often higher than that in the healthy group, suggesting great responsiveness to immunosuppressive therapy (**Figure 3C**) (19, 63). Another study demonstrated that the thickness of EOMs showed a strong correlation with cross-sectional area but a weak correlation with muscle volume, indicating that the measurement of EOM volume could not be replaced by thickness simply (64).

Classically, the volume of EOMs can be obtained by multiplying the sum of the cross-sectional areas by the layer thickness (65). Nowadays, similar to the fat volume, EOM volume can also be measured by 3D reconstruction (39) (**Figure 2**). Increased EOM volume is positively correlated with the GO severity and may contribute to optic neuropathy (56, 66). However, it remains inconclusive in GO activity (21, 38, 67, 68). One possible reason is that the enlargement of EOMs usually occurs earlier than obvious symptoms, which further highlights the role of imaging in early diagnosis.

### Inflammation evaluating in EOMs

#### T2 relaxation time (T2RT)

EOMs usually appear edematous, and the inflamed portion may produce high signals on T2WI, which has been used to assess the activity of GO. Nowadays, quantitative MRI is available. This gives a higher accuracy compared to qualitative MRI. T2 mapping is a technique to construct a map based on the T2 value calculated for each voxel. The T2 value is defined as the time until T2 has decayed to 37% of the post-excitation transverse magnetization according to the curve acquired from several single-shot images. This provides a quantitative parameter that describes the T2 signal (69). The T2RT reflects the water content of the tissue and is used as a way to assess the degree of inflammatory edema (70). This has been widely used in inflammation-related diseases such as myocardial edema, arthritis, and axial spondyloarthropathies (71–73). Likewise, T2RT of EOMs tends to increase in patients in the active phase and is positively correlated with CAS scores (27). Furthermore, T2RT showed a good prediction of the prognosis after immunosuppression therapy. Tachibana et al. found the coincidence rate of diagnosis by CAS and T2RT was relatively low (54.2%). Even in the CAS negative group, more than half had a prolonged T2RT and showed improvement after immunosuppressive therapy (43). Zhai and colleagues (20) divided patients into two groups according to the therapeutic effects of glucocorticoids. They found mean T2RT of EOMs is higher in responsive group as an independent predictor of prognosis, with area under the curve (AUC) = 0.764.

#### Fat suppression (FS) sequences

Although T2RT provides reliable information on parameters to indicate the degree of inflammation, measurement of T2RT must be accompanied by appropriate post-processing. In addition, the signal of adipose tissue is high on both T1 weight-images (T1WI) and T2 weight-images (T2WI), which can confound the water signal (74). FS sequences can suppress such fat signals to some degree, with negligible effects on water signals, allowing a better differentiation between adipose tissue and inflammatory edema (**Figures 3D, E**). Under these conditions, it was demonstrated that measurements of the signal intensity (SI) could be directly used to estimate the degree of inflammation. Commonly used fat suppression technologies include Chemical Shift-selective Fat Suppression (CHESS), short inversion time inversion recovery (STIR), spatial spectral pulse, and Dixon (75). Hoh and colleagues (76) measured signal intensities of the EOMs in 19 patients with Graves’ ophthalmopathy by STIR sequences, showing that the temporalis muscle was structurally similar to the EOMs, with little inflammation occurring in GO. Therefore, they calculated the signal intensity ratio (SIR) of EOMs and temporalis muscles as being higher in GO patients than in healthy controls and positively correlated with Werner activity scores (76). Subsequently, other researchers also evaluated the SIR and activity scores in GO patients by STIR, confirming good agreement despite the different scoring criteria in these investigations (22, 77, 78).

Dixon is often used as a T2-weighted processing technique that can directly distinguish between fat and water signals, and Dixon-T2WI suffers from fewer artifacts than STIR sequences, making it quite suitable for head and orbital imaging. Except for a water map which is equal to the fat-suppressed sequences, Dixon-T2WI can also generate a fat map, allowing for quantitative analysis of the fat content. In some studies, investigators compared Dixon-T2WI with conventional T1WI, T2WI, and other FS sequences such as fat-sat. With higher signal values in the edematous fraction, Dixon-T2WI was shown to improve the sensitivity and specificity of the diagnosis (23, 24). Accordingly, the predictive performance of treatment response by FS sequence is better than T2RT (**Figure 3F**) (18, 20).

#### Diffusion-weighted imaging (DWI)

DWI is based on the different ability of water molecules to specifically move in different tissues. If their motility decreases, the signal intensity determined by DWI increases, and vice versa, thereby exploiting regional differences in tissue-specific diffusion capacity to produce contrast. This allows the use of the apparent diffusion coefficient (ADC) to describe the extent to which water molecules are confined in different tissues (79). The degree of diffusion sensitization is described by the b-value. Higher b-values correlate with diffusion effects positively and thus more pronounced signal attenuation, but this comes with increased noise, reducing the overall signal-to-noise ratio. It is important to optimize the SNR at each b-value for multiple b-values in DWI acquisition. DWI has been widely applied in distinguishing benign and malignant tumors of, for instance, the brain, liver, lung, kidney, and other parenchymal organs, and for the identification of acute cerebral infarction and showed great sensitivity and specificity (80). The ADC also shows superiority in the observation of orbital tissue lesions and inflammation (81–83).The value can also be used to quantify the degree of inflammation in EOMs. In one study, it was shown that ADC values of the four EOMs increased sequentially when comparing a healthy control group with, respectively, a GO-uninvolved group and a GO-involved group. Consequently, DWI might identify the inflamed EOMs earlier than other sequences as it can better distinguish less advanced GO cases from healthy controls (84).

There are two major techniques for DWI available, of which the most commonly used is echo planar imaging (EPI), but it suffers from low signal-to-noise ratio and is easy to form artifacts (85). The non-EPI alternative was initially used for the diagnosis of middle ear cholesteatoma, where it resulted in fewer artifacts and a higher resolution and could identify microscopic lesions as small as 2 mm, which is better than EPI for skull base imaging (86, 87). Recently, the effect of the non-EPI alternative technique for DWI was evaluated for GO, and such studies demonstrated good results for patients with active GO and optic neuropathy (26, 88).

#### Fat infiltration in EOMs

Fat infiltration in EOMs is often observed in MR images and is probably correlated with the severity of GO. The MRI signal of the EOMs was found to be slightly lower than normal signals in the FS sequence, indicating fat infiltration in EOMs (89, 90), but this method can only be used for qualitative diagnosis. The fat fraction (FF) is a semiquantitative parameter of fat measurement, represented by the ratio of fat to the sum of water and fat in the EOMs obtained by post-processing of the data with defined calculation methods. It has been widely used to estimate fat infiltration, including vertebral tumor progression, and to evaluate surgical effects (91–93). The FF of EOM increases with the course of GO, which is consistent with its pathological process (27, 61), but the relationship between FF and the various stages of the disease is unclear. This may be related to the relatively small size of EOMs compared to other organs or tissues. The effect of edema remains large, despite an increase in fat content, causing the values of FF to fluctuate without consistent positive or negative correlation with CAS scores.

It cannot be neglected that CT is another modality to estimate fat infiltration of EOMs based on Hounsfield units (HU). The density ranges were set at −200 to −30 HU for fat, −30 to +100 HU for EOMs, and 0 HU for edema, which is sufficient to decrease the error from other infiltrations, including hyaluronic acid and lymphocytes (94, 95). However, two studies showed inconsistent results: Regensburg et al. found there was no statistical significance in the mean identity of EOMs between GO and controls, whereas Cohen et al. found that fat infiltration is prevalent in GO patients (94, 96). The difference between regions of interest (ROIs) selection (entirety of EOMs or parts of fat infiltration) could be an explanation. Furthermore, investigation into the comparison of MRI and CT in EOM fat infiltration is still deficient.

#### Fibrosis in EOMs

There is a broad clinical overlap between the active and fibrotic phases, and EOMs with fibrosis can be refractory to glucocorticoid therapy (97). Although the occurrence of fibrosis is insidious and difficult to detect from the clinical presentation, MRI has significant advantages in detecting tissue fibrosis. Ollitrault et al. used the area with low signal in both the Dixon T2WI water map and in T2WI as a marker of EOM fibrosis (23). Alternatively, enlargement of EOMs with normal T2RT was used as the basis for determining chronic fibrosis (98). However, these methods do not provide a quantitative determination for the degree of fibrosis, and the diagnostics based on such imaging is highly subjective, so that a clinical significance of this method is unclear. Similar to T2 mapping, T1mapping has been used as it can color-code T1-based signal intensity, allowing for better highlighting of small lesions (99). This showed good prediction and assessment of fibrosis in the heart, liver, kidney, and other tissues (100–102). When T1 mapping and Dixon T2 were evaluated for EOMs in GO with diplopia, it was found that the value of T1 could more reliably estimate the fibrosis than Dixon T2. Patients with decreased T1 values may have entered a stage of fibrosis. At this time, glucocorticoid treatment is of little use, and surgery should be considered instead (28).

**Figure 4:**
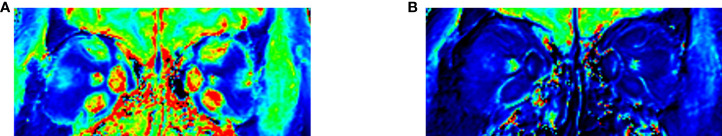
T1 mapping of EOMs. **(A)** Native T1 mapping. **(B)** Post-contrast T1 mapping for evaluating fibrosis in EOMs. Evaluating the T1 value or ECV of medial rectus and inferior rectus muscles may be sufficient for providing help for diagnosis. This figure is original.

Nevertheless, the specificity and sensitivity of non-enhanced T1mapping are not satisfied, as T1 values can also reflect the inflammation in soft tissue (103). Based on increased extracellular matrix in tissues, extracellular volume (ECV) is a derived index from pre-contrast and postcontrast T1 values that has more efficacy in detecting fibrosis (**Figure 4**). Calculation of ECV:


$$ECV=(1–\;hematocrit)\frac{1/T1(postcontrast\;rectus)-1/T1(precontrast\;rectus)}{1/T1(postcontrast\;blood)-1/T1(precontrast\;blood)}$$

A recent study indicated that one of the main pathological changes in inactive patients is muscle fibrosis. ECV rather than T1 significantly correlates with collagen volume fraction, which contributes to muscle fibrosis. This suggests that ECV may be more specific than T1 value as a parameter to assess EOM fibrosis (29).

### Lacrimal glands

#### Anatomy, histology, and pathologic change in GO

The lacrimal glands are paired amygdaloid glands located in the zygomatic process of the frontal bone. They are divided into many lobules consisting of glandular tubules and acinar portions. The interstitium of secretory tubules is scattered by lymphoid cells, mast cells, and fibroblasts, and the acinar portions are surrounded by a basal layer of myoepithelial cells (104, 105).

The surface of the lacrimal glands in GO patients expresses TSHR. Similar to the involvement of EOMs in GO, immune-related lymphocyte and monocyte infiltration can also occur in lacrimal glands (106). Inflammatory markers such as C-reactive protein, IL-1β, and IL-6 increased in the tears of GO patients, representing that the lacrimal gland is also a target organ for thyroid receptor antibody (TRAb) (107, 108). More than 30% of GO patients suffer from dry eyes, while enlarged lacrimal glands can be observed on imaging in 11% of patients (109, 110). It can even occur in cases with no change in EOMs, which may contribute to early GO detection (111).

#### MRI appearance

Trokel et al. first found lacrimal gland enlargement in GO (112). Then, several studies demonstrated objectively quantitated parameters of the lacrimal glands, such as length, width, and area, are greater in GO than healthy control. However, these morphological parameters cannot discriminate between active and inactive patients (31, 113). The volume of the lacrimal gland cannot provide additional information about diagnosis neither (114). Similar to EOMs, quantifying the degree of inflammation in the lacrimal gland may be more helpful in staging. The signal value of the lacrimal gland can also be measured on the T2 FS sequence, and the SIR can then be obtained by comparing it with the SI of the temporalis muscle. This approach can also be used as a criterion for differentiating between active and inactive GO (31). Meanwhile, the ADC and T2 values can also have a similar impact (32).

Lacrimal gland herniation is a special value characterized by Nugent et al. (115). The protrusion was determined to be at least half of the gland displaced anterior to the frontozygomatic process. This parameter was refined in subsequent studies, and reported greater bilateral lacrimal gland herniation in active GO patients than in inactive patients (30). Furthermore, compared to SIR, herniation can predict whether the patients have a response to glucocorticoids combined with orbital fat thickness (18).

Unfortunately, it seems difficult to find fibrosis in the lacrimal gland through biopsies after contrast injection (116), but the T1 value decreases more after contrast injection in active than inactive patients (32), providing a novel perspective for lacrimal gland fibrosis. It remains to be investigated whether fibrosis of the lacrimal gland has any influence on the evaluation of GO and on predicting the efficacy of glucocorticoids.

### Optic nerve

#### Anatomy, histology, and pathologic change in GO

The optic nerve extends from the retina to the brain and can be divided into the intraocular, intraorbital, intracanal, and intracranial segments. The intraorbital segment starts from the posterior of the sclera to the optic canal, represents its longest part, and is closely related to GO. It is wrapped by the optic nerve sheath, which consists of the cerebral dura mater, arachnoid mater and cerebral pia mater. The subarachnoid space of the nerve connects to the intracranial subarachnoid space and is filled with cerebrospinal fluid (117).

Dysthyroid optic neuropathy (DON), with an incidence of about 5%, is one of the most severe complications of GO (118, 119). As a result, the early detection and treatment of DON plays a crucial role in preventing permanent blindness. Several situations promote DON, including compressed optic nerves, optic neuritis, or stretched optic nerves. Over 90% of patients with DON have an enlarged EOM compressing the optic nerve, so quantification of the degree of compression helps in diagnosis. In addition, a few biopsies of nerve make optic neuritis neglected (120), but MRI may detect inflammation of the nerve, which is recommended as a typical examination for diagnosis and follow-up (121). Meanwhile, 5% of DON cases may be caused by optic nerve traction, but this mechanism is still controversial (122, 123). In conclusion, the quantitative assessment of DON by MRI focused on the size of the EOMs and optic neuritis.

#### DON prediction by MRI

Currently, there are no consistent criteria for the diagnosis of DON, which is diagnosed based on clinical symptoms such as visual impairment, visual field defects, optic disc edema, and color vision disorder (124). However, these features are non-specific and can also occur in other diseases such as idiopathic orbital inflammation or cranial nerve palsy (125). A number of ophthalmic indicators can identify subclinical DON before the onset of obvious symptoms, such as blue-yellow deficiency and thinned macular inner retina. These tests require specialized equipment and experienced ophthalmologists (126, 127). MRI, however, can clearly show the posterior state of the eye and improve the diagnosis of DON, depending on the underlying mechanism. Early studies concentrate on morphologic parameters by CT to quantify the compression. Barrett et al. defined a muscle index as a classic method by calculating the diameters of four EOMs occupied orbit (128). Weis and colleagues found that the diameter of medial muscles is suitable for predicting DON. The ROC for diagnosis was 0.83, but they did not define a specific cut-off value (66).

Rutkowska-Hinc et al. found cerebrospinal fluid in the optic nerve sheath was different between DON and non-DON patients (129). As previously mentioned, because T2 mapping and FS sequences exactly reflect the moisture content of tissues, they can be used as a new indicator for identification. Zou and colleagues modified the Barrett index by using Dixon-T2WI with higher resolution, and they calculated the index at four slices behind the eyeball. Muscle index at 21 mm combined with T2 mapping, which could indicate the rupture of the optic nerve myelin sheath and edema, improved the accuracy of diagnosis (33). On the other hand, the optic nerve subarachnoid space will increase with the edema of the optic nerve in DON. It is convenient to qualify the subarachnoid fluid volume by determining the diameters between the optic nerve sheath and the optic nerve on FS sequences and using this as a predictor (34).

## Discussion and future perspectives

In this review, we discussed comprehensive tissue-based approaches to estimating GO and provided several MRI features in different situations. We also summarized several methods for parameter measurement, but we did not provide clarity regarding how these features influence the activity phase and guide management. For example, to what extent does MRI change suggest the need for surgery or second-line therapies? Which MRI feature suggests local treatments are sufficient?

Although achievements in MRI and GO are growing rapidly, most previous studies were based on cross-sectional and retrospective analysis. Prospective studies that combine the results of treatment with multi-parameter MRI are still important. Future studies should focus on developing new sequences to improve temporal resolution, such as T1 rho, a relatively new sequence that has better characterization in injury than T1 (130). The improvement of image analysis methods for imaging, including histogram analysis, also provides more accurate ways for GO evaluation (131, 132). Furthermore, radiomics and deep learning have been widely used in image segmentation, ROI extraction, and automatic analysis to assist in diagnosis. Song and Lin et al. established two systems to discriminate GO from healthy people and detect active and inactive phases (133, 134), but investigations including larger samples and prognosis are still needed.

## Conclusion

To sum up, MRI is promising for GO assessment by providing high-resolution images and multiple functional sequences that allow physicians to intervene at the subclinical stage of GO. However, there are still some issues to be addressed, including machine diversity, time-consuming, and higher economic burden. It is crucial to establish a generally accepted test mode consisting of the necessary sequences, which is time-saving and price-friendly. This requires the coordinated efforts of endocrinologists, radiologists, and ophthalmologists.

## Data availability statement

The original contributions presented in the study are included in the article/supplementary material. Further inquiries can be directed to the corresponding authors.

## Author contributions

CS and GY wrote the manuscript. YL, HC, and JS revised the manuscript. The final manuscript was read and approved by all authors.

## Funding

This research is supported by the National Natural Science Foundation of China (82170800), the Research initiation Project of Shunde Hospital of Southern Medical University (SRSP2021001), and the Guangdong Medical Science and Technology Research Fund Project (B2022185).

## Conflict of interest

The authors declare that the research was conducted in the absence of any commercial or financial relationships that could be construed as a potential conflict of interest.

## Publisher’s note

All claims expressed in this article are solely those of the authors and do not necessarily represent those of their affiliated organizations, or those of the publisher, the editors and the reviewers. Any product that may be evaluated in this article, or claim that may be made by its manufacturer, is not guaranteed or endorsed by the publisher.

## Abbreviations

GO, Graves ophthalmopathy; GD, Graves’ disease; CAS, Clinical Activity Scores; EUGOGO, European Group on Graves’ Orbitopathy; MRI, magnetic resonance imaging; TSHR, thyroid stimulating hormone receptor; insulin-like grow factor 1 receptor (IGF-1R); OFs, Orbital fibroblasts; TH, T helper; ECM, extracellular matrix; CT, computed tomography; T1WI T1 weight-images; T2WI T2 weight-images; T2RT, T2 relaxation time; SI, signal intensity; CHESS, Chemical Shift-selective Fat Suppression; STIR, short inversion time inversion recovery; SIR, signal intensity ratio; ADC, apparent diffusion coefficient; EPI, echo planar imaging; FF, fat fraction; Hu, Hounsfield units; ROIs, regions of interest; ECV, extracellular volume; TRAb, thyroid receptor antibody; DON, dysthyroid optic neuropathy.
